# Thank you to our reviewers – 2025

**DOI:** 10.1093/hropen/hoag022

**Published:** 2026-04-18

All of the *Human Reproduction Open* team are acutely aware of the importance of high-quality peer review and its role in maintaining and enhancing the scientific quality and clinical relevance of the articles published in the journal. As a new journal, we are particularly grateful to the colleagues who have given the benefit of their expertise and dedication and helped *Human Reproduction Open* to become the well-established journal it is today. Thank you.

Abbott, David H

Acien, Maribel

Alcazar Zambaro, Juan Luis

Alteri, Alessandra

Alviggi, Carlo

Ammar, Omar

Amorim, Christiani

Anckaert, Ellen

Anifandis, George

Ata, Baris

Aurrekoetxea-Casaus, Maite

Babayev, Elnur

Baev, Vesselin

Balasubramanian, Ravi

Baldi, Elisabetta

Ben-Rafael, Zion

Bermejo-Alvarez, Pablo

Bertolla, Ricardo

Besser, Andria

Bishop, Cecily

Boiani, Michele

Bosch, Ernesto

Boudry, Liese

Brian, Kate

Buratini, Jose

Cacciottola, Luciana

Canis, M

Canosa, Stefano

Carmona, F David

Castelluccio, Noemi

Castillo, Catherine

Cavoretto, Paolo

Chang, Wei Fange

Chaube, Shail

Cheng, Lin

Cheng, Tuck Seng

Chian, Ri-Cheng

Chou, Ya-Ching

Copp, Tessa

Corti, Laura

Courtoy, Guillaume

Cozzolino, Mauro

Critchley, Hilary

Cui, Linlin

Dai, Yongdong

Datta, Adrija Kumar

De Geyter, Christian

De Neubourg, Diane

de Ziegler, Dominique

Del Gallego, Raquel

D’Hooghe, Thomas

Di Simone, Nicoletta

Donatti, Lillian

Donnez, Jacques

Dörk-Bousset, Thilo

Drakopoulos, Panagiotis

Dumont, Ludovic

Dwyer, Andrew

El Hajj, Nadi

Elkhatib, Ibrahim

Errazuriz, Joaquin

Exacoustos, Caterina

Ezoe, Kenji

Feichtinger, Michael

Felberbaum, Ricardo

Feng, Qian

Fernandez-Fuertes, Beatriz

Ferrero, Simone

Fitzgerald, Oisin

Freis, Alexander

Fu, Yonglun

Galvao, Antonio

Gardner, David

Gautier, Thomas

Giles, Juan

Goisis, Alice

Gotte, Martin

Gottert, Ann

Gravholt, Claus H

Griesinger, Georg

Gris, Jean-Christophe

Gurung, Shanti

Habiba, Marwan

Hanevik, Hans

Harada, Miyuki

Hee Lee, Seok

Heindryckx, Björn

Hilgers, Teresa

Hill, Christopher

Holt, William V

Hong, Xiang

Hossay, Camille

Hu, Jingmei

Hu, Liang

Huang, Bo

Huppertz, Berthold

Ince, Onur

Iwase, Akira

Jayaprakasan, Kannamannadiar

Jensen, Rikke Beck

Jezek, Davor

Jordan, Philip

Jukic, Anne Marie

Kamath, Mohan

Kandari, Sayali

Kang, Hang

Kao, Shu-Huei

Karaer, Abdullah

Katsika, Evangelia

Kaya, Cihan

Kelleher, Andrew

Khambata, Kushaan

Kovacic, Borut

Ku, Seung Yup

Kumar Adiga, Satish

Kunicki, Michał

Kurimoto, Kazuki

Kyhl, Frederik

Laan, Maris

Labrosse, Julie

Laurentino, Sandra

Lawrenz, Barbara

Leandri, Roger Dominique

Lee, Tsung-Hsien

Lee, Yi Xuan

Legro, Richard

Li, Fei

Li, Mengying

Li, Shumin

Li, Wentao

Liao, Tierong

Lin, Chih-Wan

Liu, Kimberly

Liu, Xitong

Liu, Yanhe

Luddi, Alice

Lundy, Scott D

Luo, Qiong

Luque, Guillermina

Lydic, Michael

Maclean, Alison

Makieva, Sofia

Makinen, Sirpa

Malek, Janet

Marin, Diego

Marklund, Anna

Martinez, Francisca

Martins, Wellington

Masciangelo, Rossella

Massarotti, Claudia

McElreavey, Kenneth

McNamara, Blair

Mechsner, Sylvia

Melton, Phillip E

Mijatovic, Velja

Mitchell, Rod

Miyashita, Mariko

Moghetti, Paolo

Mol, Ben

Molinari, Emanuela

Molinas, Carlos

Morin-Papunen, L

Morris, Joan

Mustaniemi, Sanna

Narasimhan, Kothandaraman

Neuhaus, Nina

Nezhat, Ceana

Nordhoff, Verena

Nosrati, Reza

Oberle, Anna

Oktem, Ozgur

Olszewska, Marta

Orvieto, Raoul

Ota, Kuniaki

Pabuccu, Emre

Pachernegg, Svenja

Paffoni, Alessio

Pauli, Andrea

Peate, Michelle

Pedro, Juliana

Perrin, Jeanne

Perrotta, Manuela

Perruzza, Davide

Polyzos, Nikolaos

Pool, Thomas

Priskorn, Laerke

Provoost, Veerle

Quartucci, Antonio

Quinton, Richard

Racowsky, Catherine

Raja, Edwin Amalraj

Ramalho-Santos, João

Reis, Fernando

Roberts, Stephen

Roelens, Caroline

Romito, Alessia

Rönö, Kristiina

Ross, Whitney Trotter

Salumets, Andres

Sangalli, Juliano Rodrigues

Santos-Ribeiro, Samuel

Schallmoser, Andreas

Schmidt, Lone

Sefrioui, Omar

Seljeflot, Eline Bragedatter

Selntigia, Aikaterini

Shikanov, Ariella

Sibiude, Jeanne

Silva Gallardo, Constanza

Siristatidis, Charalampos

Skedgel, Chris

Smeenk, Jesper

Somigliana, Edgardo

Sonigo, Charlotte

Spaan, Mandy

Steeper, Michelle

Stern, Judy

Stojanov, Milos

Subiran, Cristina

Subramanian, Anita

Sun, Fei

Sun, Yuelian

Takahashi, Toshifumi

Tapanainen, Juha

Taylor, Tyl

Tempest, Nicola

Tierney, Katherine

Töyli, Mira

Tros, Rachel

Tsaltas, Jim

Tulandi, Togas

Tyc, Katarzyna

van Duursen, Majorie

Vanni, Valeria

Vassard, Ditte

Vazquez-Levin, Mónica

Verpoest, Willem

Viganò, Paola

Vigneswaran, Kugajeevan

Vitale, Francisco

Vomstein, Kilian

von Versen-Höynck, Frauke

Vriens, Joris

Wehrli, Lydia

Westvik-Johari, Kjersti

Williams, Carrie

Williams, Zev

Wong, Chris K C

Wyrwoll, M J

Xiong, Jiaqiang

Xu, Hong

Yahubyan, Galina

Yan, Junhao

Yang, Qian

Yang, Xiaoyu

Yassa, Murat

Yeung, William S B

Yildiz, Sule

Zacharin, M R

Zambelli, Fillipo

Zedeler, Anne

Zeng, Lin

Zhang, Junyu

Zhu, Yeyi

Zizolfi, Brunella

